# Local photo-mechanical stiffness revealed in gold nanoparticles supracrystals by ultrafast small-angle electron diffraction

**DOI:** 10.1063/1.5091858

**Published:** 2019-04-12

**Authors:** Giulia Fulvia Mancini, Francesco Pennacchio, Tatiana Latychevskaia, Javier Reguera, Francesco Stellacci, Fabrizio Carbone

**Affiliations:** 1 Laboratory for Ultrafast Spectroscopy, Lausanne Center for Ultrafast Science (LACUS), École Polytechnique Fédérale de Lausanne, CH-1015 Lausanne, Switzerland; 2 Paul Scherrer Institut, OVGA/319, 5232 PSI Villigen, Switzerland; 3 Laboratory for Ultrafast Microscopy and Electron Scattering, Lausanne Center for Ultrafast Science (LACUS), École Polytechnique Fédérale de Lausanne, CH-1015 Lausanne, Switzerland; 4 Physics Institute, University of Zurich, Winterthurerstrasse 190, 8057 Zurich, Switzerland; 5 CIC biomaGUNE, Paseo de Miramón 182C, 20014 Donostia-San Sebastian, Spain and Ikerbasque, Basque Foundation for Science, 48011 Bilbao, Spain; 6 Supramolecular Nanomaterials and Interfaces Laboratory, Institute of Materials, École Polytechnique Fédérale de Lausanne, CH-1015 Lausanne, Switzerland

## Abstract

We demonstrate that highly ordered two-dimensional crystals of ligand-capped gold nanoparticles display a local photo-mechanical stiffness as high as that of solids such as graphite. In out-of-equilibrium electron diffraction experiments, a strong temperature jump is induced in a thin film with a femtosecond laser pulse. The initial electronic excitation transfers energy to the underlying structural degrees of freedom, with a rate generally proportional to the stiffness of the material. Using femtosecond small-angle electron diffraction, we observe the temporal evolution of the diffraction feature associated with the nearest-neighbor nanoparticle distance. The Debye-Waller decay for the octanethiol-capped nanoparticle supracrystal, in particular, is found to be unexpectedly fast, almost as fast as the stiffest solid known and observed by the same technique, i.e., graphite. Our observations unravel that local stiffness in a dense supramolecular assembly can be created by van der Waals interactions up to a level comparable to crystalline systems characterized by covalent bonding.

Two-dimensional supracrystals created by self-assembly of nanoparticles (NPs) offer a route toward engineering materials with specific functionalities.^1,2^ The building blocks are metallic core NPs functionalized with a ligand shell of organic molecules, which determine the macroscopic structural, electronic, optical, and magnetic properties of these systems.^3–6^ In alkanethiol-protected gold NP supracrystals, the competition between thermodynamic driving forces can lead to structural phases ranging from crystalline to glassy by simply changing the length of the alkanethiol molecules.^7^ The structural diversity and the stabilization of these supracrystals are primarily affected by the level of ligand interdigitation.^8,9^

Simultaneous characterization of the NP cores and their ligand shell, as well as of the short-range properties of the NP assembly, has proven to be challenging so far.^1^ Mueggenburg *et al.* showed that monolayers of dodecanethiol-capped gold nanoparticles display remarkable properties of mechanical strength, comparable to those of glassy polymers, accompanied by robustness and resilience at higher temperatures.^10^ In this work, however, macroscopic (i.e., long range) properties of the overall colloidal crystal were analyzed. To circumvent the lack of direct experimental observation of the ligand structures in alkanethiol-capped gold NP supracrystals, Salerno *et al.* performed molecular dynamics (MD) simulations. Their results show that variations of the ligand structure directly correlate with the membrane stiffness, with the retrieved Young's moduli being related to the nanoscale ligand structural features.^11,12^

In alkanethiol-capped NP colloidal crystals, the local (i.e., short-range) mechanical properties are primarily affected by the local arrangement of NP cores and organic ligands within single grains. For this, a scattering technique is required, which can combine the angstroms (Å) spatial resolution with sensitivity to light elements. Effects of the ligand chain length on NP packing density or disorder have recently been explored in two-dimensional ligand-stabilized NP supracrystals by Kim *et al.,*^7^ where equilibrated monolayers were produced by cyclic compression and relaxation in Langmuir trough, and their phase transition from crystalline to liquid through a hexatic phase was interpreted as the entropy-driven phenomenon associated with steric constraints between ligand shells.

Only recently, ultrafast small-angle electron diffraction has enabled us to resolve both their static ordering properties and their photo-induced motions^13^ with the combined femtosecond (fs) temporal and Å spatial resolution. In this work, we observe that the light-induced decay of the intensity of the diffraction feature associated with the local (nearest to the next-nearest neighbor) hexagonal arrangement of the gold NPs in each supracrystal depends on the length of the functionalizing ligands. For the shortest ligands, we find that the rate of this decay is as fast as what is observed in a very stiff solid such as graphite, characterized by strong homonuclear covalent bonding. The transient response from supracrystals of dodecanethiol-coated gold nanoparticles, instead, is found to be significantly slower, comparable to softer systems. Our experimental results are supported by simulations, which demonstrate that the local symmetry of the NPs within the supracrystal grains affects the short-range degree of coupling between the electronic and lattice degrees of freedom. Our novel approach allows us to open a viewpoint on a topic in which only speculations or *ab initio/semi-empirical* calculations were possible so far and offers a unique experimental observation for describing collective modes in soft matter, a topic of great fundamental and applied interest.

We conducted experiments on three different 2D nanoparticle supracrystals. Each sample consists of monodisperse gold NPs of the same size (∼5 nm) coated with different alkanethiols (R−SH, R = C_n_H_2n+1_): 1-octanethiol (n = 8), 1-dodecanethiol (n = 12), and 1-octadecanethiol (n = 18). Within the text, we will refer to these samples as C8, C12, and C18, respectively. Each NP supracrystal monolayer was obtained by Langmuir−Schaefer deposition^13–15^ (the details on the synthesis are available in the supplementary material). The supracrystals' time-dependent response is probed using small-angle ultrafast electron diffraction in transmission geometry, with the diffraction patterns forming onto a phosphor screen and detected using a single-electron counting charge-coupled device. The experiments were conducted with consistent parameters for all three samples. At every time delay, a single image is the result of 500 accumulations with 300 gates per exposure. The diffraction patterns were acquired an overall current of 320.4 pA for every time delay on each sample.

The total charge amount, distributed over several electron pulses, was calculated taking into account the number of accumulations/time delays and the total number of time delays, and was estimated to be 0.25 *μ*C, 0.411 *μ*C, and 0.23 *μ*C for the C8, C12, and C18 samples, respectively. Photoinduced changes in the samples were initiated by 1.5 eV pump-pulses focused to a spot of 220 *μ*m. The incident fluence on the sample was 13 mJ/cm^2^ (100 mW at 20 kHz with the spot size of 220 *μ*m). The effectively absorbed fluence was estimated around 100 *μ*J/cm^2^, based on the optical reflectivity of gold in a layer of 7 nm thickness, the sample density, and the penetration depth of gold for electrons, (7–8 nm at 1.5 eV). Experiments were conducted at room temperature in transmission geometry with an almost collinear arrangement between the pump and probe pulses. The background pressure in the experimental vacuum chamber was ∼10^−9^ mbar. A schematic of our apparatus is available in Fig. 1 and in Refs. 13 and 16.

Structure retrieval methods based on the calculation of the angular Cross-Correlation Function (CCF) have proved effective in retrieving the local arrangement and the symmetry of the NPs in the supracrystal.^13^ In our experiment, this information is contained in the small-angle region of the diffraction patterns from each supracrystal, at the scattering vector *q*_1_ marked in the inset of Fig. 1. For each sample, the diffraction feature at the scattering vector *q*_1_ is related to the real space distance *d*_1_ of crystallographic planes created by nanoparticles in the supracrystal [Fig. 2(b)]. The values of *q*_1_ for each sample are reported in Table I. As mathematically derived in Ref. 17, characteristic symmetries in such dense systems of identical particles can be detected in the CCF when the coherence length of the probing wave is at least comparable to the size of the single particle. The normalized CCF is defined as^18–23^

$$C_{\mathrm{norm}}\left(\Delta \right)=\frac{{\left\langle I\left(q_{1},\varphi \right)I\left(q_{1},\varphi +\Delta \right)\right\rangle }_{\varphi }-\;{{\left\langle I\left(q_{1},\varphi \right)\right\rangle }^{2}}_{\varphi }}{{{\left\langle I\left(q_{1},\varphi \right)\right\rangle }^{2}}_{\varphi }},$$ (1)

where $I(q_{1},\varphi )$ represents the scattered intensity at the scattering vector *q*_1_ and the angle φ, *Δ* is the shift between the two angles (inset of Fig. 1), and ${\left\langle \right\rangle }_{\varphi }$ denotes an averaging over φ. Following the approach described in Ref. 13, the normalized CCF at the scattering vector *q*_1_ for each sample was obtained from the one-dimensional Fourier spectrum of the scattered intensity $I(q_{1},\varphi )$. The details on this analysis are available in the supplementary material.

**FIG. 1.**
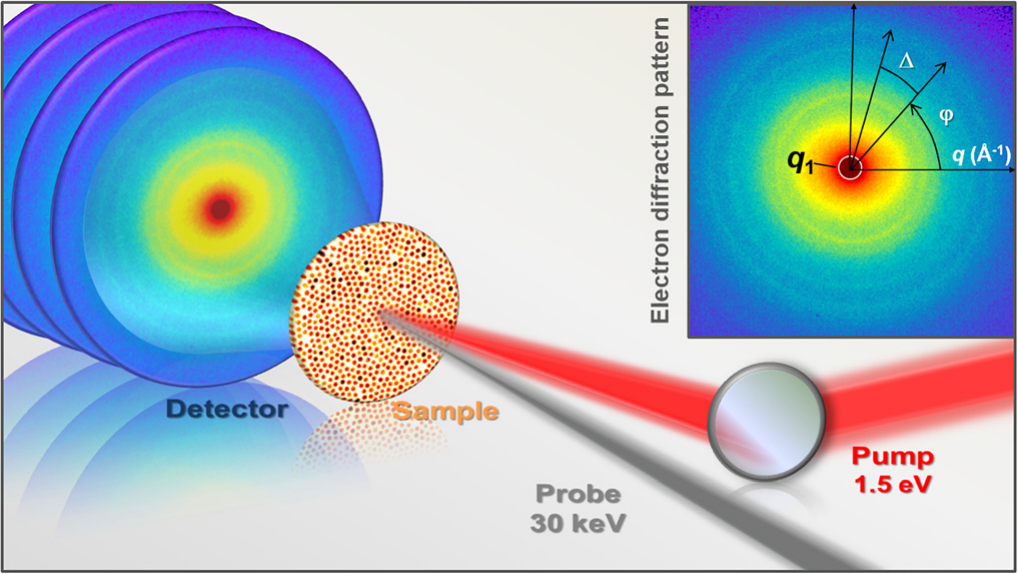
Ultrafast small-angle electron scattering experimental layout. Inset: electron diffraction pattern and illustration of the concept of angular cross-correlation analysis.

**FIG. 2.**
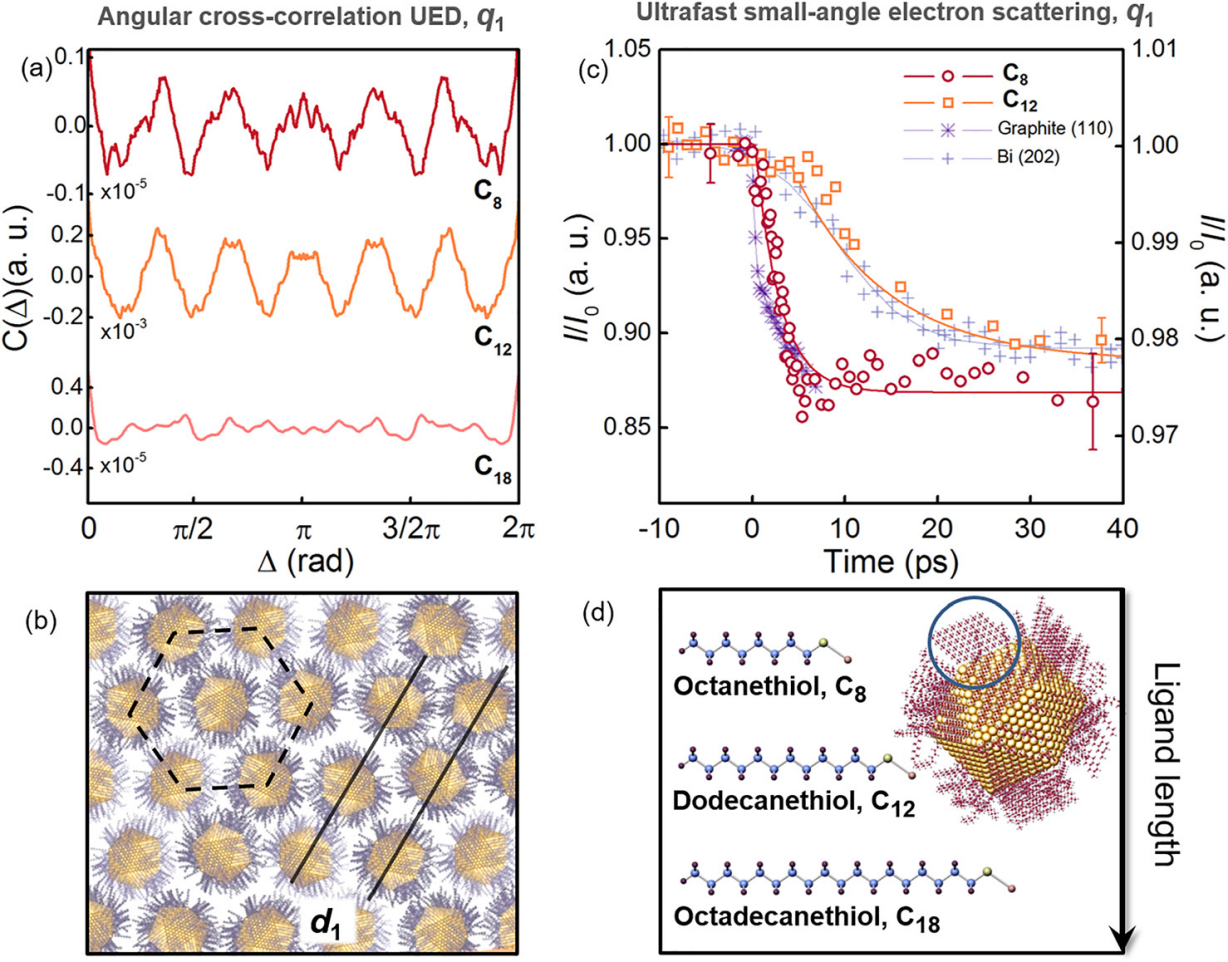
Ligand length-dependent dynamic response of C8 and C12 supracrystals upon photoexcitation. (a) Angular cross-correlation analysis reveals a hexagonal arrangement of the NPs in the C8 (red) and C12 (orange) supracrystals and the liquid-like phase of the C18 (pink) sample. (b) Schematic illustration of a two-dimensional monolayer of alkanethiol-functionalized NPs. The NPs are arranged in a hexagonal lattice and create crystallographic planes, which distance *d*_1_ is primarily a function of the ligand length. (c) NP dynamics for the C8 (red circles) and C12 (orange squares) supracrystals, compared to the time response of graphite,^25^ and bismuth data.^26^ In this panel, the transient change in $I\left(q_{1}\right)$ for the C8 (red circles) and the C12 (orange squares) supracrystals is reported as I, and I/I_0_ indicates that $I\left(q_{1}\right)$ for each sample was normalized to its average value at negative times (*t* < *t*_0_). All experimental time traces were fitted to a mono-exponential curve (solid lines). (d) Simulation of a single thiolated NP and comparative display of ligand structures and the length (Table I).

**TABLE I.** Relevant distances and time scales for the C8, C12, and C18 supracrystals.

Sample	*q*_1_ (Å^–1^)	Ligand length (nm)	Core-core distance (nm)	Time scale *τ* (ps)
C_8_	0.118	1.285	6.1	2.6 ± 0.3
C_12_	0.101	1.789	7.2	12.1 ± 0.9
C_18_	0.098	2.542	7.4	…

Figure 2(a) displays the CCFs retrieved at equilibrium (i.e., before photoexcitation) for the C8 (red), C12 (orange), and C18 (pink) supracrystals. The 6-fold modulation of the CCF from the C8 and C12 samples reflects the hexagonal close-packed arrangement of the NPs in the supracrystals [Fig. 2(b)]. This suggests the presence of a crystalline structural phase in which neighboring NPs are held together within single grains by attractive van der Waals forces that lead to the favorable interdigitation of the ligands.^7–9^ Thus, NPs in each grain arrange in crystallographic-like planes with the distance of $d_{1}=\frac{2\pi }{q_{1}}$ [Fig. 2(b)]. The absence of recognizable symmetries for the C18 sample indicates a lack of short-range order in the NP self-assembly, likely due to repulsive forces winning over van der Waals attractive interactions,^7^ as well as to lower NP solubility and mobility at room temperature. A simulation of a single alkanethiol-coated NP is reported in Fig. 2(d). Gold NP cores of ∼5 nm are simulated as having a polyhedral morphology, with the gold atoms arranged in a face-centered cubic (fcc) lattice.^13,24^ The chemical structure of each ligand (n = 8, 12, and 18) is displayed for clarity. The ligand lengths and the average core-core NP distances for each supracrystal, retrieved experimentally with electron diffraction, are summarized in Table I.

We analyzed the transient changes in the NP hexagonal arrangement at *q*_1_ for the C8 and the C12 supracrystals and compared their time responses [Fig. 2(c)]. The radial average intensity at *q*_1_ is calculated as

$$I\left(q_{1}\right)=\frac{1}{2\pi }\int I(q_{1},\varphi )d\varphi .$$ (2)

The transient change in $I\left(q_{1}\right)$ for the C8 (red circles) and the C12 (orange squares) supracrystals is reported in Fig. 2(c), where I/I_0_ indicates that $I\left(q_{1}\right)$ for each sample was normalized to its average value at negative times (*t* < *t*_0_). All experimental time traces were fitted to a mono-exponential curve (solid lines). Photoinduced thermal disorder in the NP hexagonal arrangement is evidenced in both samples by the transient decrease in $I\left(q_{1}\right)$. Remarkably, the $I\left(q_{1}\right)$ decay time scale for C8, τ = 2.6 ± 0.3 ps, is significantly shorter than the one for C12, τ = 12.1 ± 0.9 ps.

The $I\left(q_{1}\right)$ suppression for C8 and C12 is due to the energy transfer between the electronic excitation made by light and the underlying structural degrees of freedom of the supracrystal. In Fig. 2(c), both decay traces are compared with the ones detected in transmission ultrafast electron diffraction in two solid state systems characterized by a vastly different electron-phonon coupling strength, namely, graphite^25^ (purple asterisks) and bismuth^26^ (grey crosses). In nanostructured systems such as the ones investigated in this work, the presence of attractive van der Waals interactions among ligands should act as a glue to hold the NPs in the supracrystal together via interdigitation. Such non-covalent “bonding,” and its dynamical response to energy transfer, should lead to a disordering of the NP local arrangement on time scales comparable if not slower to those of a soft solid, such as bismuth. While our observations suggest that this scenario applies for the C12 supracrystal, where the relaxation follows a time scale τ = 12.1 ps, the dynamics observed in the C8 supracrystal is dramatically different and unexpected.

In the C8 supracrystal, the intensity drop of the diffracted beam is characterized by a Debye-Waller factor comparable to the one of graphite, which is to date the fastest ever observed in ultrafast electron diffraction.^25,27,28^ In graphite, which microscopic structure is characterized by the presence of strong homonuclear covalent bonds, a bi-exponential decay of $I\left(q_{110}\right)$ revealed the presence of strong coupling between the electronic subset with a small subset of lattice degrees of freedom (τ = 250 fs), followed by carriers cooling through electron-phonon and phonon-phonon scattering with a time scale of τ = 6.5 ps.^25^ The comparable rapidity in the suppression of the diffracted intensity for C8 suggests that in this supracrystal, the interdigitation of the shorter ligands provides a very efficient channel for transferring energy between the initial electronic excitation and structural motions of the NPs.

Our results can be rationalized following two main arguments. First, we consider that upon photoexcitation of a metallic system, the evolution of the electronic temperature and lattice temperature can be described via the popular two-temperature model,^29^ in which hot electrons exchange energy with the phonons sub-system. Consequently, such a sub-system increases its temperature. The speed of this energy transfer is directly reflected by the evolution of the Debye-Waller effect in a diffraction experiment, as the phonon excitation disorder atomic position, thus affecting the diffraction intensity.^27,30,31^ In more complex solids, it is possible that only a sub-set of phonons is excited first^27,31–33^ or that other bosonic subsystems can drain the excess energy from the hot electrons. In cuprate superconductors, for example, a four-temperature model was proposed to account for the energy transfer between hot electrons, phonons, and spin fluctuations.^34,35^ Despite the rich variety of circumstances that one can encounter, it is always true that the speed of the electronic or the lattice temperature decay is directly related to both the coupling between the initial excitation and the modes transferring the excess energy and their own energy.

Furthermore, recent MD simulations^11,12^ were carried out on periodic dry membranes of 6 × 6 NPs, thus approximating the arrangement of NPs within single supracrystal grains, to which our experiments are sensitive. These theoretical results demonstrated a direct correlation between the supracrystal local stiffness and the ligand structure, thereby predicting our experimental observations at the nearest neighbor level. At a microscopic level, the description of the collective modes responsible for the observed energy transfer between the electronic excitation and soft-matter supracrystals is a subject of current investigation, leading to the development of exciting new concepts such as the existence of short-range phonons in soft matter and liquids, also termed anakeons.^36^

As demonstrated by the CCF static analysis [Fig. 2(a)], the distribution of NPs in the C18 sample is the one of a glassy phase. Thus, we cannot identify a distinct diffraction feature to follow its temporal evolution. For this reason, the temporal dependence of the scattered intensity $I\left(q_{1}\right)$ for the C18 supracrystal is not reported in Fig. 2(c). The photoinduced disorder observed in both C8 and C12 supracrystals is accompanied by annealing of NPs grains, evidenced by a transient increase in the signal-to-noise ratio of the CCFs at different time delays (see supplementary material). This behavior, already reported for the C12 supracrystal,^13^ is now visualized also in the C8 sample and indicates that local order is impulsively triggered, within the relevant time scales, in supracrystals where the NP distribution is stabilized by ligand interdigitation.

Next, we demonstrate for each sample the presence of a direct correlation between the observed Debye-Waller decay and the symmetry of the NPs within the supracrystal grains. Ligand length-dependent order-disorder correlations in the three supracrystals were explored in a series of simulations. The Transmission Electron Microscopy (TEM) images of the C8, C12, and C18 samples measured using UED are displayed in Fig. 3. The corresponding diffraction patterns are simulated as the squared amplitude of the Fourier transform (FT) of the TEM images (the details are provided in the supplementary material). Each two-dimensional Fourier transform is normalized to the number of nanoparticles detected in the corresponding TEM image. The patterns in Fig. 3 directly visualize a hexagonal order at *q*_1_ in the C8 and C12 samples. The core-core distance values extracted from the FTs of the TEM images are in agreement with the values retrieved experimentally with electron diffraction (Table I). For the C18 supracrystal, the observed Airy pattern is created by the diffraction on randomly distributed objects, i.e., the NPs, with an aperture diameter equal to the diameter of the NP. The Airy pattern indicates a glassy structural phase of the C18 supracrystal, which was experimentally demonstrated using CCF analysis [Fig. 2(a)]. The average of the NPs in C18 was estimated to be 5.4 nm by fitting the radial averaged intensity $I\left(q\right)$ with the following function:

$$I\left(q\right)={\left(\frac{J_{1}\left(\frac{\pi \mathrm{qd}}{\lambda z}\right)}{\left(\frac{\pi \mathrm{qd}}{\lambda z}\right)}\right)}^{2},$$ (3)

with *J*_1_ being the Bessel function of the first kind, *d* the NP diameter, *q* the abscissa coordinate, *λ* the wavelength, and *z* the sample-detector distance.

**FIG. 3.**
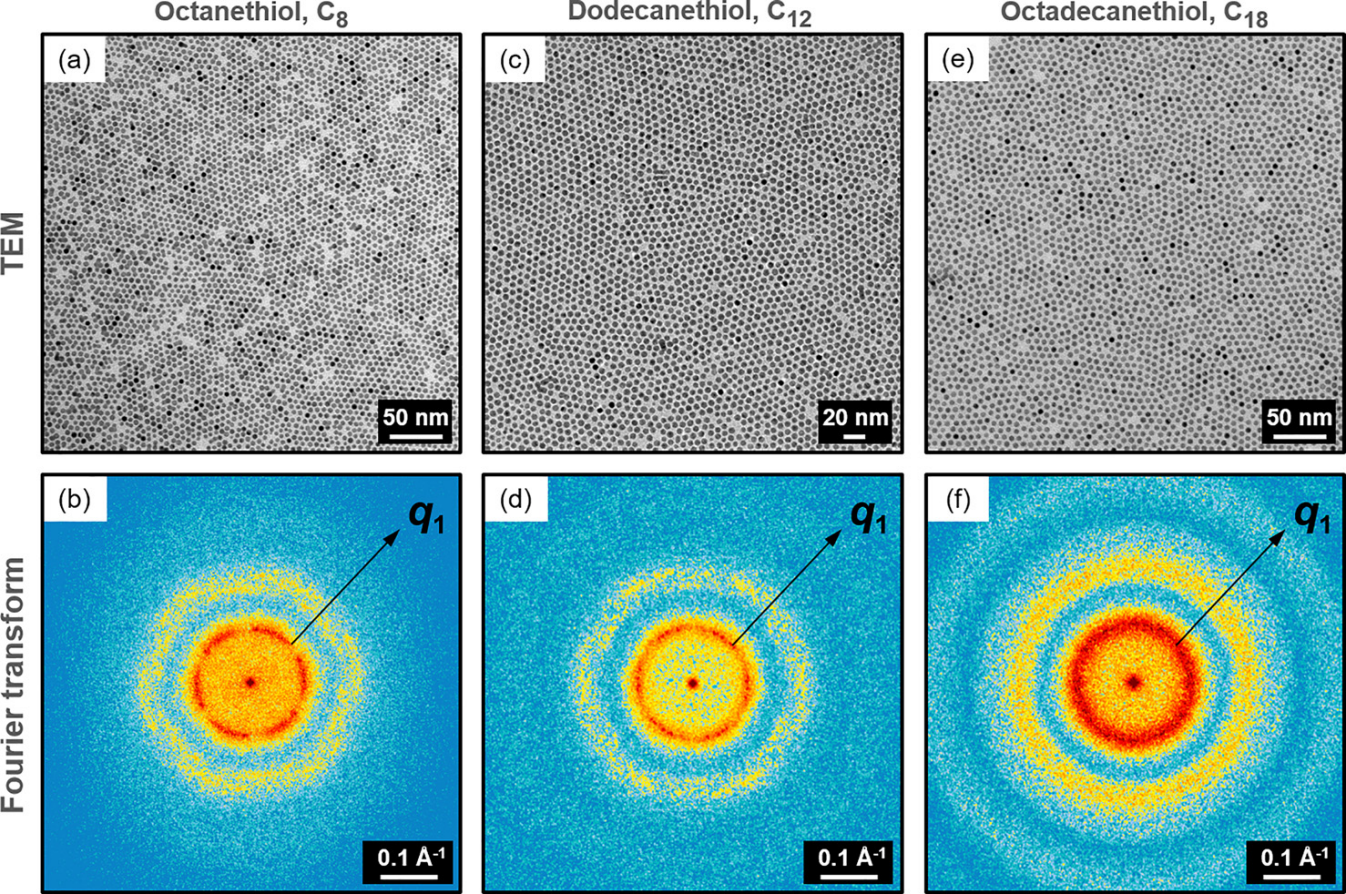
Experimental TEM images and Fourier transforms. (a), (c), and (e) TEM images of the C8, C12, and C18 supracrystals (left to right) measured using UED. (b), (d), and (f) diffraction patterns simulated as the squared amplitude of the Fourier transform of the TEM images in (a), (c), and (e), respectively.

Sphere lattice model (SLM) simulations are used to assign the different local distribution of the NPs within supracrystal grains (Fig. 4). The models are created from a two-dimensional lattice of opaque spheres of ∼5 nm diameter, arranged in a hexagonal lattice. Round domains are selected to have a size of 60 nm and a centre-to-centre distance between domains of 80 nm. The rotation angle of the domains is Gaussian distributed with the mean = 0 and standard deviation σ. Two distributions are shown in Fig. 4: (i) σ = 10° with a perfectly ordered spheres arrangement preserved within each domain (left) and (ii) σ = 10° with disorder of individual NP positions introduced by adding a random shift up to Δ*r* = ±1 nm (right).

**FIG. 4.**
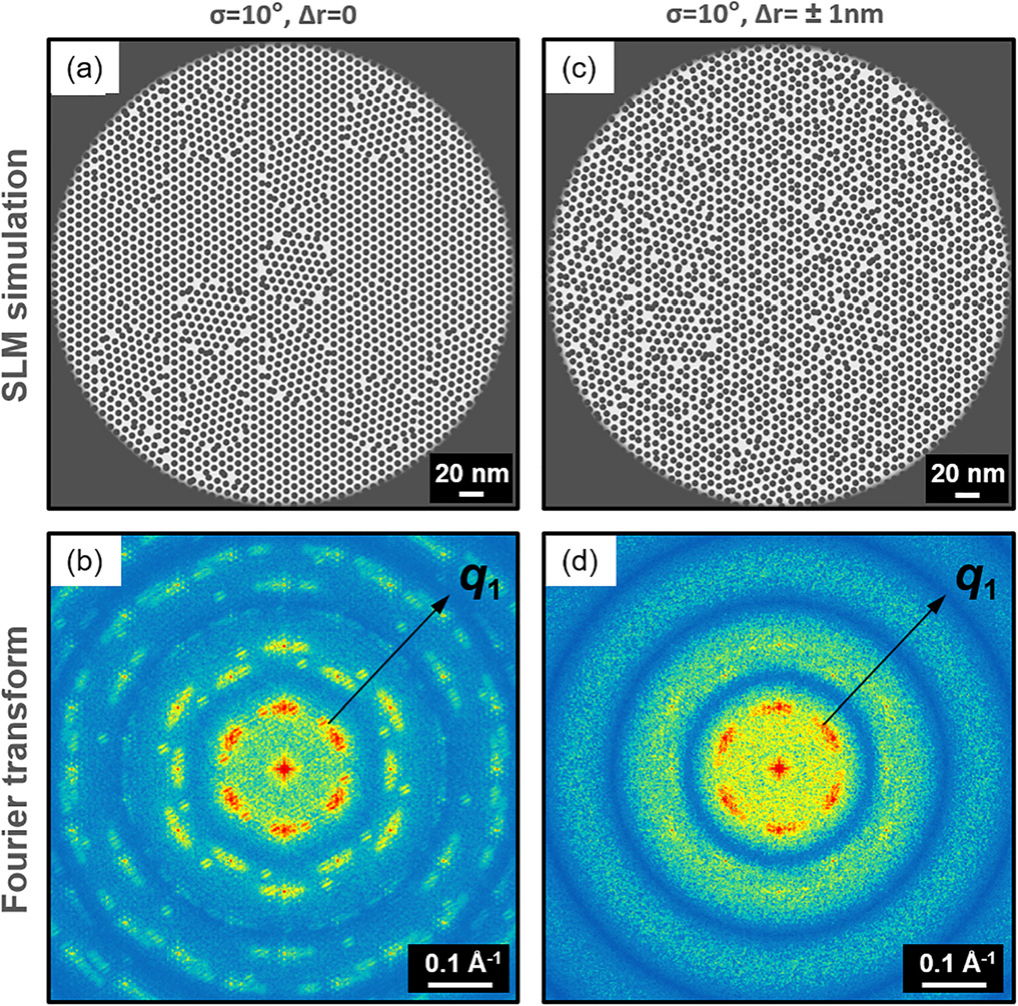
Simulations of the TEM images considering each NP as a spherical opaque object. (a) σ = 10° with a perfectly ordered sphere arrangement preserved within each domain. (b) Fourier transform of (a). (c) σ = 10°, with disorder of individual NP positions introduced by adding a random shift up to Δ*r* = ±1 nm. (d) Fourier transform of (c).

The location of *q*_1_ is indicated in Fig. 3 for the experimental and Fig. 4 for the simulated Fourier transforms. In each, *q*_1_ marks the first order of diffraction from the crystallographic planes with distance *d*_1_ displayed in Fig. 2(b). The results show a remarkable agreement between (i) the [10°, 0] model with the C8, C12 samples and (ii) the [10°, ±1 nm] model with the C18. The similarity between (i) the [10°, 0] SLM model with the C8 and C12 samples and (ii) the [10°, ±1 nm] SLM model with the C18 is confirmed by the radial averaged intensity plots in Figs. 5(a) and 5(b). Multiple orders of diffraction from the *q*_1_ crystallographic planes are observed in systems characterized by the presence of local symmetry (C8, C12). These diffraction orders progressively smear into a pattern from chaotically distributed objects by increasing the randomization in the NPs positions (C18).

**FIG. 5.**
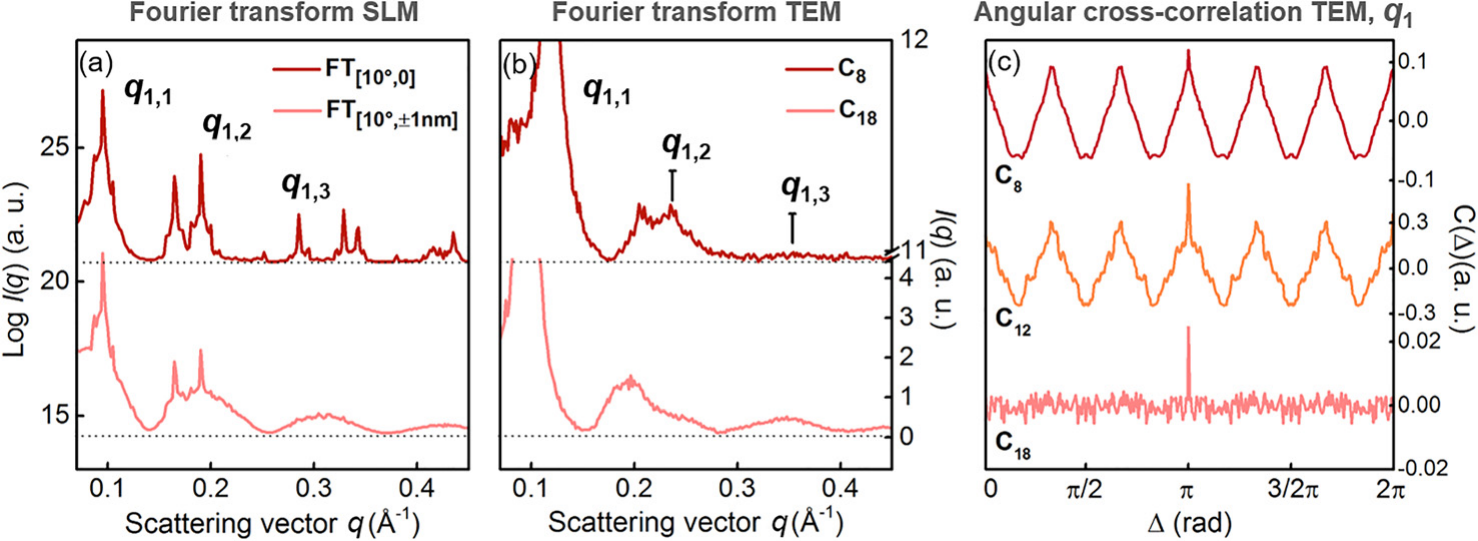
Compared intensity profiles from experimental and simulated Fourier transforms and angular cross-correlation analysis of the C8, C12, and C18 TEM images. (a) Radial averaged intensity of the Fourier transform of the [10°, 0] simulation (red) and of the [10°, ± 1 nm] simulation (pink). (b) Radial averaged intensity of the Fourier transform of the TEM images for C8 (red) and C18 (pink). (c) Angular cross-correlation functions at *q*_1_ from the Fourier transform of the TEM images of the C8 (red), C12 (orange), and C18 (pink) samples. The location of *q*_1_ in the two-dimensional simulation is reported in Figs. 3(b), 3(d), and 3(f).

In the C8 and C12 supracrystals, where a transient suppression of $I\left(q_{1}\right)$ is observed, the simulations confirm a direct connection between the observed photo-mechanical local stiffness with the presence of a recognizable translational symmetry of the NP distribution within single domains. In C18, where no local symmetry is found, the randomized distribution of the scattering objects within each domain leads to the presence of a liquid-like phase, where no correlation between transient changes and NP arrangement can be unraveled. These observations are supported by the analysis of the CCFs retrieved from the Fourier transforms of the TEM images in Fig. 5(c). The minor differences (for example, in contrast at *q*_1_) between the FTs from TEM experimental images (Fig. 3) and the FTs from the corresponding SLM simulations (Fig. 4) are solely due to a different degree of disorder of spheres and to the lack of random additional noise in the SLM simulated data, as confirmed by the full agreement between the CCFs at *q*_1_ of the experimental data [Fig. 2(a)] and of the FTs of TEM experimental images [Fig. 5(c)].

In this work, we reported that local mechanical stiffness can be created in supramolecular assemblies by van der Waals forces to an extent comparable to systems characterized by strong covalent bonding. The key to our observation is a powerful technique that combines small-angle electron diffraction (Å spatial resolution) with the ultrafast (fs) temporal resolution. Moreover, we demonstrate that the local symmetry of the NPs within the supracrystal grains affects the short-range degree of coupling between the electronic and lattice degrees of freedom. We point out that our small-angle scattering technique is sensitive to the short-range arrangement of a few nearest-neighbors (alkanethiol-capped gold NPs) within supracrystal grains, in samples that are overall characterized by the presence of discontinuities, as dislocations and grain boundaries. Thus, the transient response observed in each sample unveils information about photo-mechanical local stiffness, i.e., at the nearest/next nearest neighbor level. Because of the presence of defects and dislocations in the overall colloidal crystal, we remark that the observed stiffness is not expected to directly translate into a macroscopic mechanical property. Our results provide a seed for new theoretical models of structural collective modes in soft-matter systems.

## SUPPLEMENTARY MATERIAL

See supplementary material for experimental design, sample preparation, cross-correlation functions, Fourier transform of TEM images and simulations, azimuthal intensity and Fourier spectrum, and supracrystal transient annealing.
